# Clinical outcomes of over-the-scope-clip system for the treatment of acute upper non-variceal gastrointestinal bleeding: a systematic review and meta-analysis

**DOI:** 10.1186/s12876-019-1144-4

**Published:** 2019-12-23

**Authors:** Chunyu Zhong, Shali Tan, Yutang Ren, Muhan Lü, Yan Peng, Xiangsheng Fu, Xiaowei Tang

**Affiliations:** 1 Department of Gastroenterology, Affiliated Hospital of Southwest Medical University, Street Taiping No.25, Region Jiangyang, Luzhou, 646099 Sichuan Province China; 2 Departmemt of Gastroenterology, Beijing Tsinghua Changgung Hospital, School of Clinical Medicine, Tsinghua University, Beijing, China; 3 Department of Gastroenterology, Affiliated Hospital of North Sichuan Medical College, Road Wenhua 63#, Region Shunqing, Nanchong, 637000 Sichuan China

**Keywords:** Endoscopic, Gastrointestinal bleeding, Over-the-scope clip, Systematic review, Meta-analysis

## Abstract

**Background:**

Conventional endoscopic treatments can’t control bleeding in as many as 20% of patients with non-variceal gastrointestinal (GI) bleeding. Recent studies have shown that over-the-scope-clip (OTSC) system allowed for effective hemostasis for refractory GI bleeding lesions. So we aimed to conduct a systematic review to evaluate the effectiveness and safety of the OTSC system for management of acute non-variceal upper GI bleeding.

**Method:**

A comprehensive literature search was conducted on PubMed, EMBASE, and Cochrane Library covering the period from January 2007 to May 2019. The literature was selected independently by two reviewers according to the inclusion and exclusion criteria. The statistical analysis was carried out using Comprehensive Meta-Analysis software version 3.0.

**Results:**

A total of 16 studies including 769 patients with 778 GI bleeding lesions were identified. Pooled technical success was achieved in 761 lesions [95.7%; 95% confidence interval (CI), 93.5–97.2%], and the pooled clinical success was achieved in 666 lesions (84.2, 95% CI, 77.4–89.2%). The incidence of re-bleeding was reported in 81 patients and the post-procedure mortality was 10.9% (*n* = 84). Only 2 (0.3%) patients occurred complications after OTSC system procedure.

**Conclusions:**

Our study demonstrated that the OTSC system was a technically feasible modality and highly efficacious in achieving hemostasis in acute non-variceal upper gastrointestinal bleeding.

## Background

Upper gastrointestinal bleeding is preponderantly non-variceal in origin and remains one of the commonest challenges faced by endoscopists in daily clinical practice. It is estimated that the incidence of annual acute upper non-variceal gastrointestinal bleeding (UNVGIB) ranges between 50 and 160 cases per 100,000 and commonly requires hospitalization [1]. Despite crucial advances in the treatment of UNVGIB over the past decade, including optimal use of endoscopic therapy and high-dose proton pump inhibition, UNVGIB still carried considerable morbidity, mortality and health economic burden [2]. The majority of UNVGIB could be managed by conventional endoscopic interventions. However, some studies have shown that conventional therapies such as epinephrine injections and hemoclips or coagulation could not achieve successful hemostasis in 4 to 20% of UNVGIB cases, and even caused severe complications [3–8].

Hence, there was an urgent need for a safe and more effective endoscopic treatment modality for UNVGIB cases. Recently, a novel endoscopic device, called Over-the-scope clip (OTSC) system has been developed. The OTSC system is a clipping device made of nitinol, and easily attached to the tip of the scope. By a procedure similar to ligation of esophageal varices, it can be readily released at the site of the bleeding site. Since the first report of the successful application of OTSC in GI bleeding by Kirschniak et al. in 2007 [9], there have been many prospective or retrospective studies reported OTSC system to manage the UNVGIB. Thus, we aimed to perform a systematic review and structured meta-analysis of all eligible studies to evaluate the effectiveness and safety of OTSC system in patients with UNVGIB.

## Methods

### Search strategy

This meta-analysis was conducted in accordance with Preferred Reporting Items for Systematic Reviews and Meta-Analyses (PRISMA) guidelines [10]. For this topic, the participants were the patients with acute upper non-variceal gastrointestinal bleeding, the intervention was a treatment method of OTSC, and the outcome was the success rate.

Studies published in PubMed, Embase and Cochrane library from January 2007 to May 2019 was searched systematically using the following search terms, “OTSC system”, “over the scope clip”, “OVESCO”, “gastrointestinal bleeding”, “ulcer bleeding”, “melena”, “hemorrhage”, “hemostasis” and others. The terms were used in all possible combinations to obtain the maximal number of articles. Additional file 1: Table S1 showed the search strategy of each of search engines. The identified studies were subsequently screened for duplicates and relevance on the subject by their abstracts. Two reviewers independently searched literature and reviewed of the identified studies for eligibility. If there was any disagreement, it could be resolved through discussion between the two reviewers or judged with the assistance of a third party.

### Criteria for inclusion and exclusion

Studies reporting primary data in which UNVGIB was managed with OTSC system were included. Essential results were defined as following: primary hemostasis defined as no re-bleeding immediately after OTSC placement. Primary failure defined as continuous bleeding after OTSC placement. Re-bleeding was defined as development of fresh hematemesis, melena, hematochezia, shock, or a drop in hemoglobin of more than 2 g/dL within 24 h, with need for repeat treatment [11–13]. Technical success defined as successful placement of the OTSC on the target lesion. Clinical success defined as having no primary failure and no re-bleeding during follow-up. The above results must be reported in included studies. Only studies in English were included. And series with more than 5 cases described were included. Vivo trials were excluded. Chronic bleeding was excluded as well.

### Data extraction

The following data were extracted from each study: (a) study characteristics, including the author name, the publication country, publication year, type of study, sample size, age, gender, lengths of follow-up, and (b) clinical features of use OTSC system managing bleeding lesion, including indication, bleeding classification, number of patients receiving an antithrombotic, technical success, clinical success, re-bleeding, number of OTSC system deployments, number of blood units transfusion, number of patients requiring additional surgical procedures, complications and mortality.

### Study quality

Downs and Black checklist were used to evaluate the quality of the included studies [14]. We scored each study in accordance with the evaluation scale, high methodological quality articles were defined as scoring higher than 19, moderate quality articles as scoring 15 to 19, moderate to low-quality articles as scoring 10 to 14, and low quality articles as scoring lower than 10.

### Statistical analysis

Continuous data are presented as the mean ± standard deviation (SD) or variation range. The statistical analysis was carried out using Comprehensive Meta-Analysis software version 3.0. The overall success rate of clinical trials was expressed by the pooled proportion with 95% confidence intervals (CI), which was presented as forest plots. Statistical heterogeneity of included studies was evaluated using Cochran Q test and *I*^2^ statistic, *I*^2^ value of greater than 50% or a *P* value of less than 0.05 for the Q statistic was considered to indicating significant heterogeneity. Then, the pooled proportion used a random-effects model or fixed-effects model. Publication bias was assessed via visual inspection of the funnel plot and Egger’s test, the *P* value of greater than 0.05 was considered to no publication bias [15].

## Results

### Included studies

**Fig. 1:**
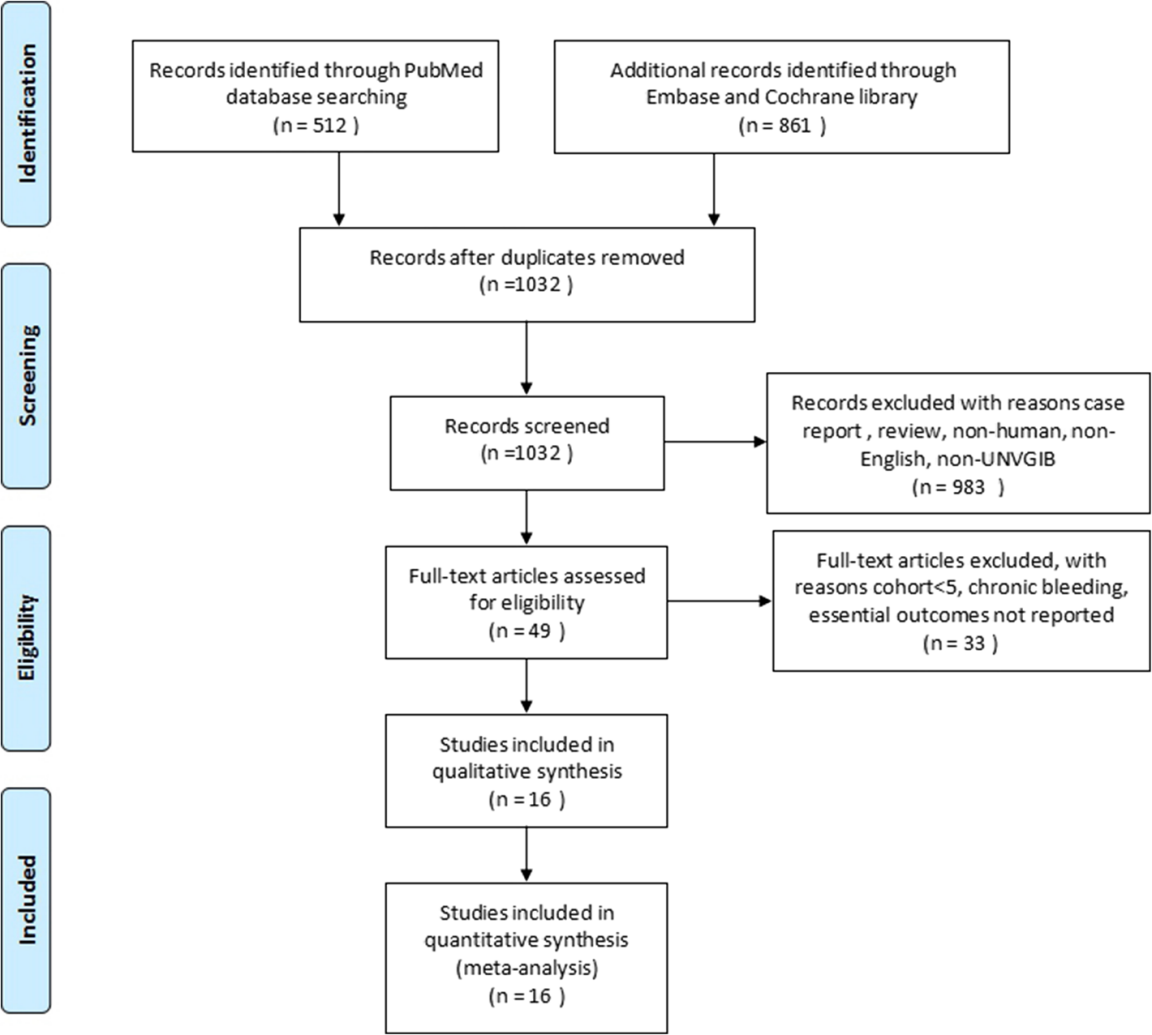
Flowchart for search strategy and selection of eligible studies

The initial search terms identified 1032 studies after duplicates removed. Of these, 983 studies were excluded according to the predefined criteria and 49 studies were reviewed the full-text. Final literature search and selection according to the inclusion and exclusion criteria yielded 16 studies about OTSC system treatment for UNVGIB [11–13, 16–28]. Figure 1 showed the search and selection process.

### Studies characteristics

Studies characteristics are summarized in Table 1. All the studies were published between 2011 and 2019, and most studies published in Germany (*n* = 7). Only one prospective randomized trial was included [26], and the remaining studies were retrospective studies (*n* = 13) or prospective studies (*n* = 2).

**Table 1:** Characteristics of included studies

Study	Country of publication	Year of publication	Study design	Patient, n	Lesion, n (P/R)	Age (years)	Gender (M:F)	Mean follow-up time (range)
Kirschniak et al. [16]	Germany	2011	Retrospective	12	12 (−/−)	–	–	–
Albert et al. [17]	Germany	2011	Retrospective	6	6 (2/4)	–	–	At least 1 month (−)
Manta et al. [18]	Italy	2013	Retrospective	23	23 (0/23)	–	–	At least 1 month (−)
Mönkemüller et al. [19]	United States	2014	Retrospective	6	6 (0/6)	72 ± 14.5	4:2	–
Chan et al. [11]	China	2014	Prospective	9	9 (3/6)	72.5 (39–89)	4:5	–
Skinner et al. [12]	United States	2014	Retrospective	12	12 (0/12)	59 (29–86)	8:4	At least 1 month (−)
Manno et al. [20]	Italy	2016	Retrospective	40	40 (40/0)	69 (25–94)	33:7	At least 1 month (−)
Wedi et al. [21]	France	2016	Retrospective	41	41 (13/28)	–	–	–
Richter-Schrag et al. [22]	Germany	2016	Retrospective	63	69 (39/30)	68 (27–92)	38:25	–
Lamberts et al. [23]	Germany	2017	Retrospective	68	68 (−/−)	–	–	–
Goenka et al. [24]	India	2017	Prospective	6	6 (0/6)	62 ± 13.1	5:1	At least 1 month (1–1.4)
Wedi et al. [25]	Germany	2017	Retrospective	118	120 (120/0)	71 ± 12.4	–	–
Schmidt et al. [26]	Germany	2018	Prospective randomized trial	33	33 (0/33)	77 (33–90)	20:13	At least 1 month (−)
Asokkumar et al. [27]	Singapore	2018	Retrospective	18	19 (10/9)	68 ± 15.9 (22–91)	12:6	At least 1 month (−)
Manta et al. [13]	Italy	2018	Retrospective	214	214 (214/0)	66 ± 10.2	115:99	At least 1 month (−)
Gölder et al. [28]	Germany	2019	Retrospective	100	100 (66/34)	76 (20–98)	64:36	–

*M:F* male to female, *P* primary treatment, *R* rescue treatment

A total of 769 patients with 778 GI bleeding lesions was managed by OTSC system. Among them, OTSC system was used as the primary treatment modality in 507 lesions, while 190 lesions as rescue treatment modality after previous endoscopic treatment failure. Only 10 studies included 501 patients reported the patients’ gender and age. Of them, 303 (60.5%) patients were male, and the mean age ranged from 59 to 77 years. Follow-up time was reported in 8 studies, with at least 1 month.

### Clinical outcomes

**Fig. 2:**
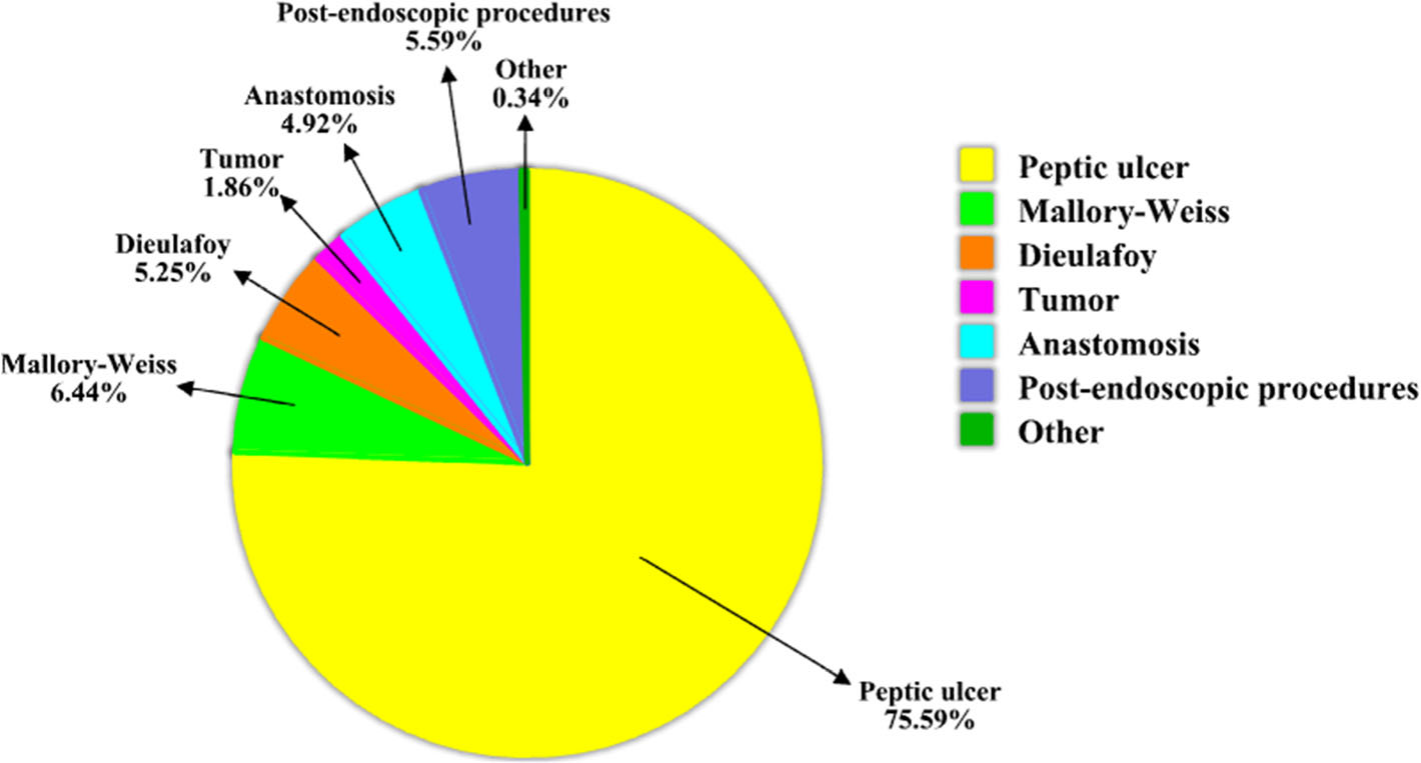
Chart for the proportion of acute upper non-variceal gastrointestinal bleeding etiology (Post-endoscopic procedures: after gastric biopsy, gastric polypectomy, endoscopic ultrasonography guided fine needle aspiration of peri-gastricmass, endoscopic mucosal resection and endoscopic submucosal dissection; Others: balloon dilation for achalasia and vascular malformation)

The etiology of acute upper non-variceal gastrointestinal bleeding was available in 14 studies. As shown in Fig. 2, the major cause was peptic ulcer (*n* = 446, 75.59%), the remain causes were Mallory-Weiss lesion (*n* = 38, 6.44%), post-endoscopic procedures (*n* = 33, 5.59%), anastomosis (*n* = 29, 4.92%), dieulafoy lesion (*n* = 31, 5.25%), tumor (*n* = 11, 1.86%) and others (*n* = 2, 0.34%). There were 7 studies reporting patients who were under the antithrombotic therapy, the proportion of which rang from 16.7 to 75.6%. The average length of hospital stays reported in 4 studies, which range from 4 to 19.8 days. Additionally, 13 studies reported 81 patients occurred re-bleeding, and the rate varied from 0 to 35.3%. The number of OTSC system deployment per lesion was reported in 8 studies, varying from 1 to 2. Six studies reported the patients received transfusion red blood cells, the mean number of blood units transfused ranged from 0 to 5.1 units (Table 2).

**Table 2:** Clinical outcomes of OTSC

Study	Indication, n	Bleeding classification (spurting/oozing/visible vessel/ adherent clot)	No. of receiving an antithrombotic	Technical success, n (%)	Clinical success, n (%)	No. of OTSC deployments, n	Re-bleeding, n (%)	Additional therapy, n (E/S/V)	Number of blood units transfused	Post-procedure 30-day mortality, n (%)	Complications of OTSC, n
Kirschniak et al. [16]	Peptic ulcer (8), Mallory-Weiss (1), Dieulafoy (1), Tumor (2)	–	–	12 (100%)	10 (83.3%)	–	2 (16.7%)	2 (2/0/0)	–	–	0
Albert et al. [17]	Peptic ulcer (5), Tumor (1)	–	–	6 (100%)	4 (66.7%)	1.17 (1–2)	2 (33.3%)	2 (0/1/1)	–	1 (16.7%) (due to multiorgan failure)	1 (leak)
Manta et al. [18]	Peptic ulcerr (18), Mallory-Weiss (2), Dieulafoy (2), Anastomosis (1)	–	–	22 (95.7%)	21 (91.3%)	1	2 (8.7%)	3 (2/0/1)	–	0	0
Mönkemüller et al. [19]	Peptic ulcer (5), Dieulafoy (1),	1/5/−/−	–	6 (100%)	6 (100%)	–	–	–	–	–	0
Chan et al. [11]	Peptic ulcer (7), Tumor (2)	–	–	9 (100%)	7 (77.8%)	–	2 (22.2%)	2 (0/1/1)	–	1 (11.1%) (due to re-bleeding)	0
Skinner et al. [12]	Peptic ulcer (8), Mallory-Weiss (1), Dieulafoy (2), Anastomotic (1)	2/6/2/1	–	12 (100%)	10 (83.3%)	–	2 (16.7%)	2 (1/1/1)	5.1 (2–12)	0	0
Manno et al. [20]	Peptic ulcer (29), Mallory Weiss (2), Dieulafoy (6), Anastomotic (3),	3/12/14/−	–	40 (100%)	40 (100%)	–	0	0	–	3 (7.5%) (related to comorbidity)	0
Wedi et al. [21]	Peptic ulcer (38), Balloon dilation for achalasia (1), Tumor (2)	9/3/23/3	31 (75.6%)	35 (85.4%)	35 (85.4%)	1.05 (1–2)	–	6 (0/6/0)	–	11 (26.8%) (5 due to re-bleeding; 6 due to other causes)	–
Richter-Schrag et al. [22]	Peptic plcer (48), Mallory-Weiss (2), Dieulafoy (4), Post-endoscopic procedures (5), Anastomoses (5), Vascular malformation (1), Tumor (4)	20/36/10/3	29 (46.0%)	68 (98.6%)	61 (88.4%)	1	–	–	–	17 (27.0%) (4 due to re-bleeding; 13 due to comorbidity)	1 (obstruction)
Lamberts et al. [23]	–	–	–	68 (100%)	44 (64.7%)	–	24 (35.3%)	–	–	–	–
Goenka et al. [24]	Peptic ulcer (4), Mallory-Weiss (1), Post-endoscopic procedures (1)	3/3/0/0	1 (16.7%)	6 (100%)	6 (100%)	1	0	0	0	0	0
Wedi et al. [25]	–	–	77 (65.3%)	120 (100%)	104 (86.7%)	1	16 (13.3%)	–	–	24 (20.3%) (7 due to re-bleeding or continued bleeding; 17 due to other cause)	–
Schmidt et al. [26]	Peptic ulcer (33)	5/18/7/3	15 (45.5%)	31 (93.9%)	28 (84.8%)	1.03 (1–2)	5 (15.2%)	2 (2/1/0)	3 (0–23)	4 (12.1%) (not related to re-bleeding)	0
Asokkumar et al. [27]	Peptic ulcer (12), Dieulafoy (4), Post-endoscopic procedures (3)	10/0/5/4	10 (52.6%)	19 (100%)	13 (68.4%)	1.10 (1–2)	0	6 (6/0/1)	1.5 ± 1.2 (0–4)	3 (17%) (due to comorbidity)	0
Manta et al. [13]	Peptic ulcer (131), Mallory-Weiss (29), Dieulafoy (11), Anastomosis (19), Post-endoscopic procedures (24)	97/117/−/−	–	208 (97.2%)	202 (94.4%)	–	9 (4.2%)	21 (−)	2 (range 1–3)	4 (1.9%) (due to re-bleeding or continued bleeding)	–
Gölder et al. [28]	Peptic plcer (100)	51/23/26/−	44 (44%)	99 (99%)	75 (75%)	–	17 (17%)	17 (6 /2/9)	3.34	16 (16%) (9 due to re-bleeding or continued bleeding, 7 due to other cause)	–

*OTSC* Over-the-Scope Clip, *E* endoscopic, *S* surgery, *V* vascular embolization, *EMR* endoscopic mucosal resection, *Post-endoscopic procedures,* after gastric biopsy, gastric polypectomy, endoscopic ultrasonography guided fine needle aspiration of peri-gastricmass, endoscopic mucosal resection and endoscopic submucosal dissection

**Fig. 3:**
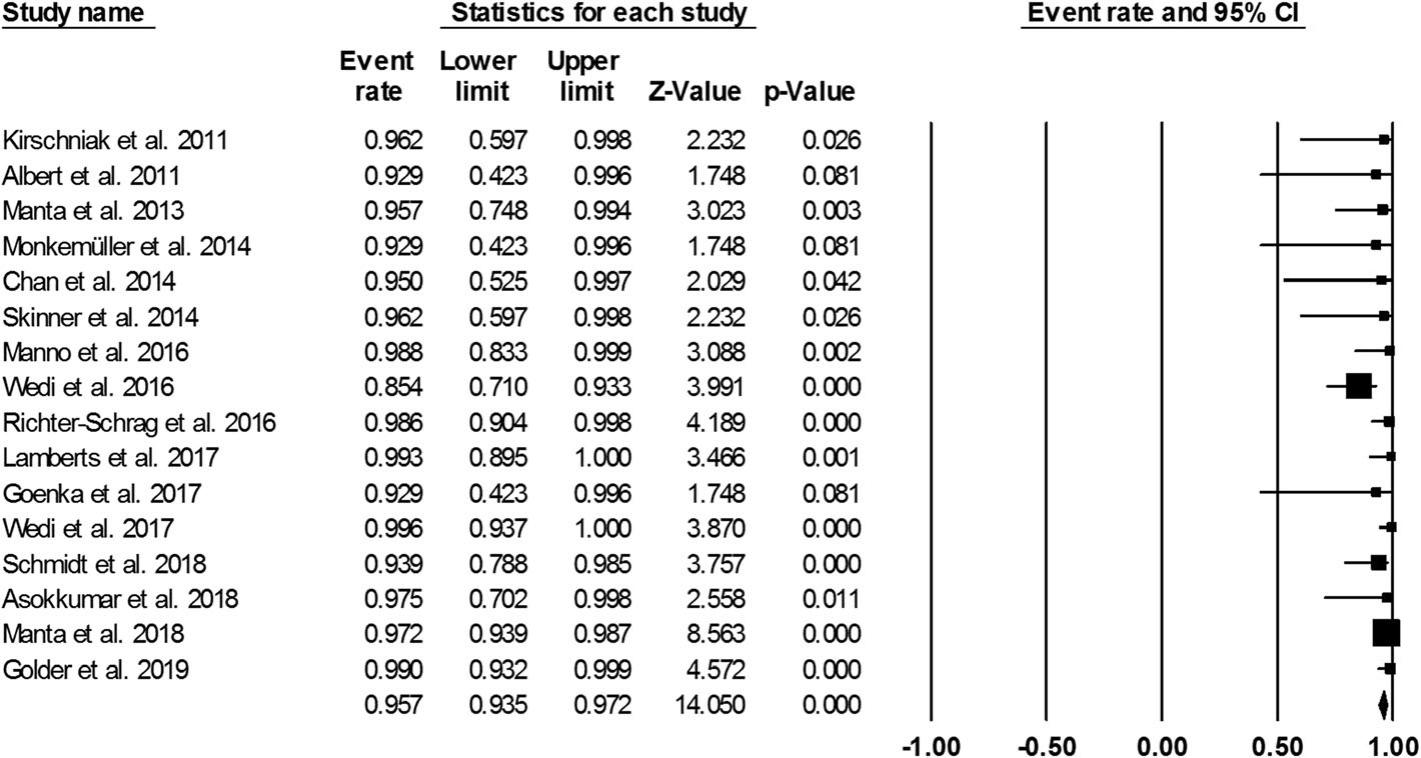
Forest plot of technical success of over-the-scope clip for acute upper non-variceal gastrointestinal bleeding

**Fig. 4:**
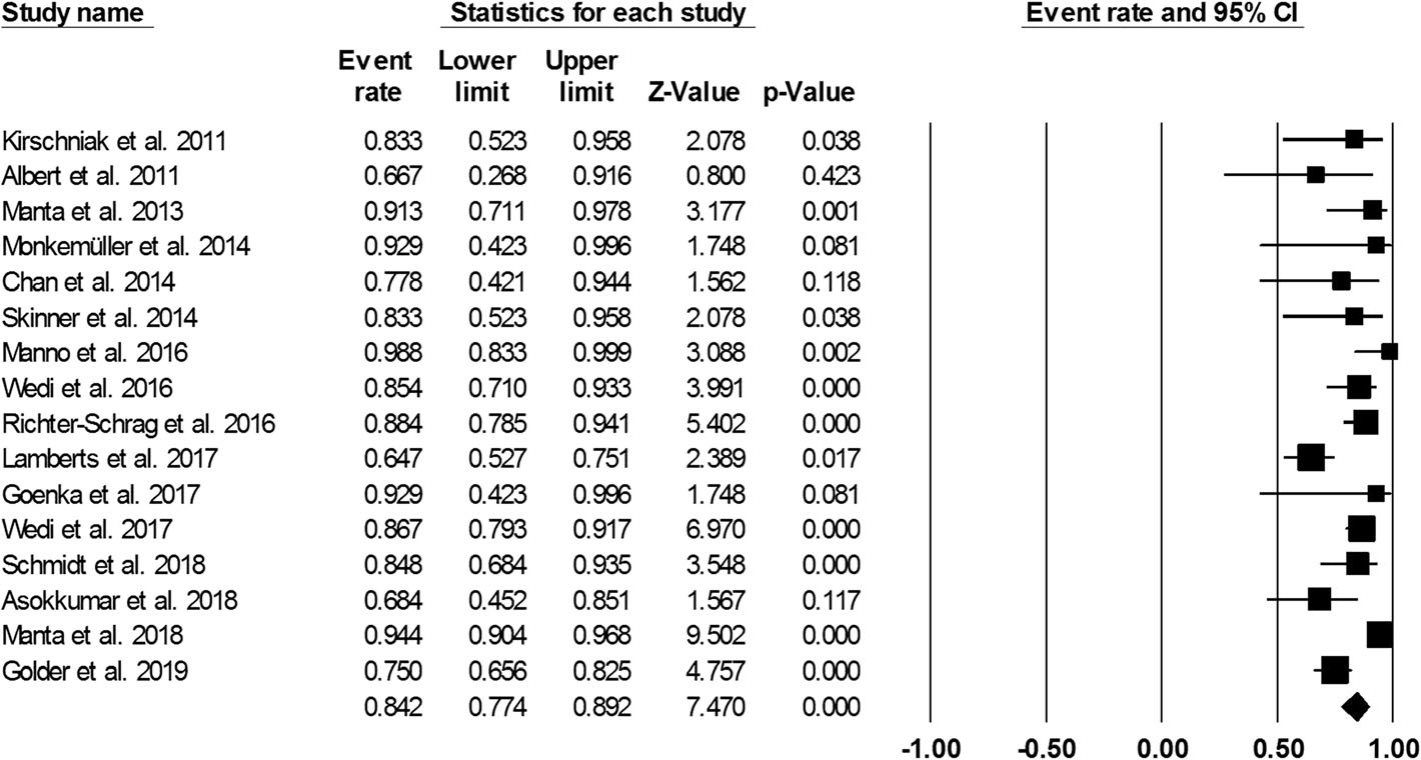
Forest plot of clinical success of over-the-scope clip for acute upper non-variceal gastrointestinal bleeding

**Fig. 5:**
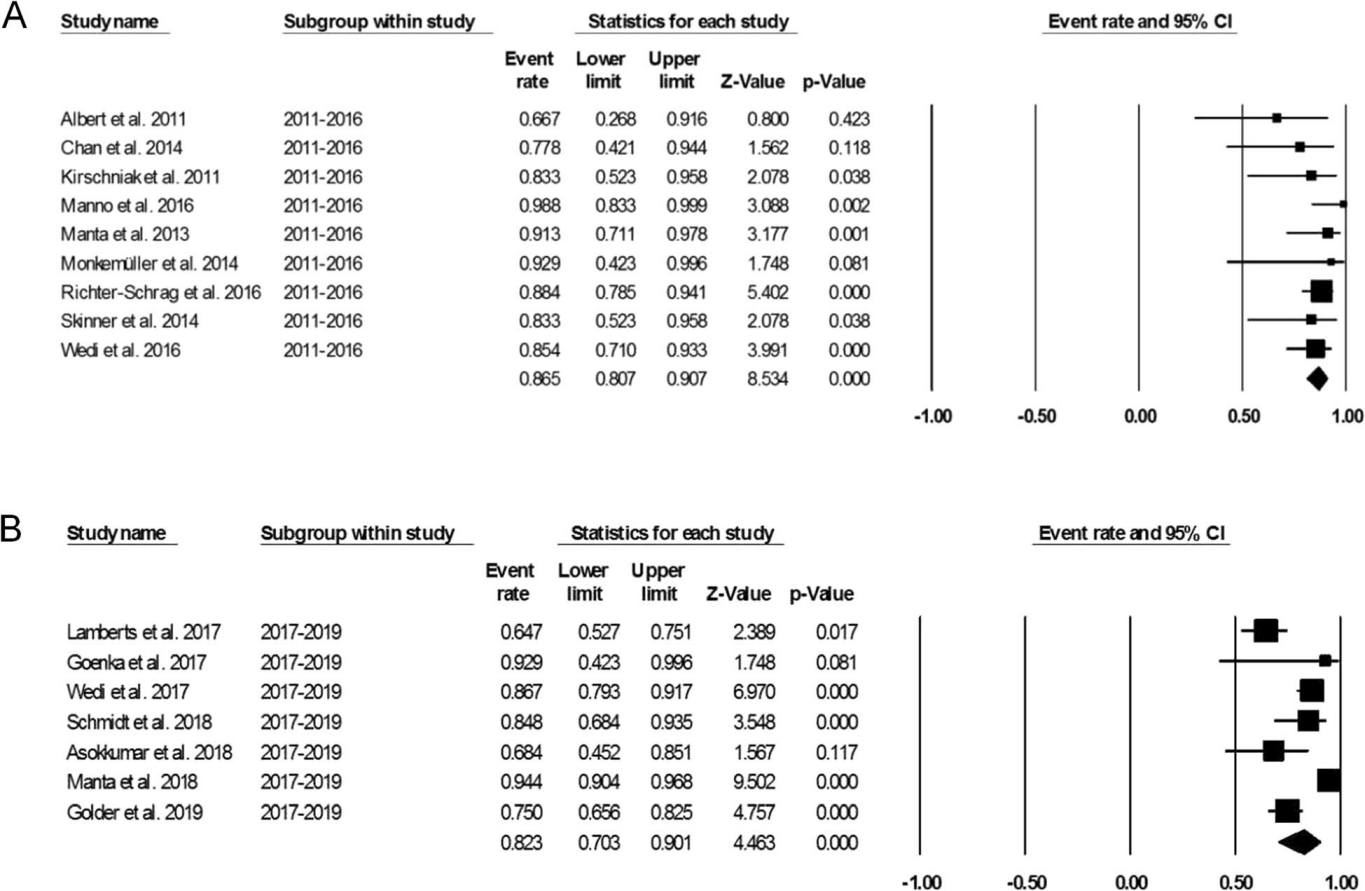
**a**. Forest plot of clinical success of studies published between 2011 and 2016. **b**. Forest plot of clinical success of studies published between 2017 and 2019

**Fig. 6:**
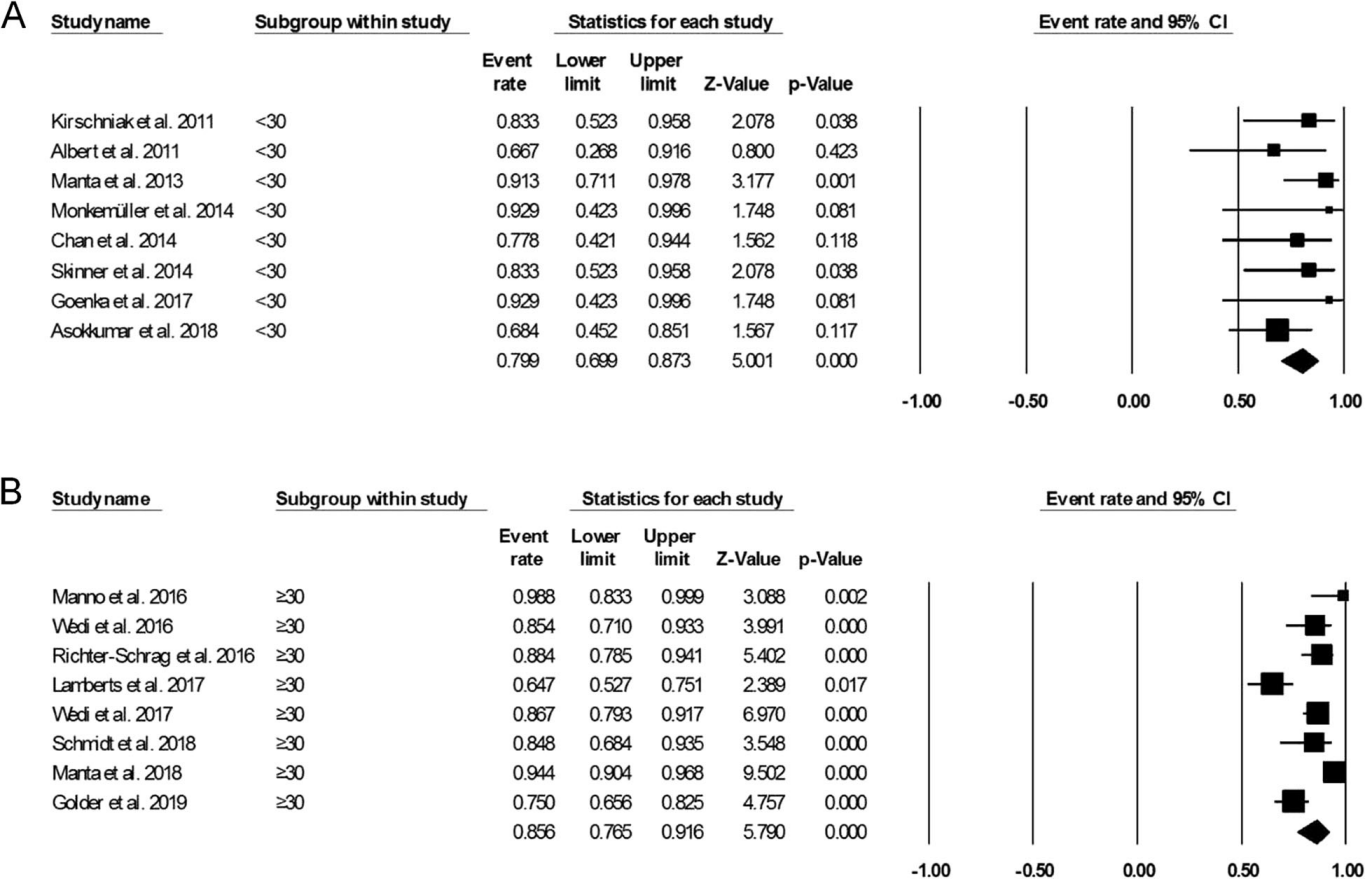
**a**. Forest plot of clinical success of studies with less than 30 patients. **b**. Forest plot of clinical success of studies with greater than or equal to 30 patients

Figure 3 showed the pooled technical success of OTSC system which was achieved in 761 lesions (95.7%; 95%CI, 93.5–97.2%). Heterogeneity was not significant among the studies (*I*^2^ = 24.5%, *P* = 0.177). Figure 4 showed the pooled clinical success was achieved in 666 lesions (84.2%; 95% CI, 77.4–89.2%). Heterogeneity was significant among the studies (*I*^*2*^ = 69.5%, *P* = 0.000). However, a total of 63 patient required additional endoscopic therapy, surgery or vascular embolization. We also conducted subgroup analysis to identify the effect of study period and study sample size in the OTSC treatment in UNVGIB. Nine studies (*n* = 218) were published between 2011 and 2016, while 7 studies (*n* = 560) were publised between 2017 and 2019. Subgroup analysis showed the clinical success rate was 86.5% (95%CI, 80.7–90.7%) and 82.3% (95% CI, 70.3–90.1%), respectively (Fig. 5). The number of studies with less than 30 patients or these with greater than or equal to 30 patients was same (*n* = 8). Subgroup analysis showed the clinical success rate was 79.9% (95% CI, 69.9–87.3%) and 86.6% (95% CI, 76.6–91.6%), respectively (Fig. 6).

### Adverse events

Most of the involved studies reported no complication occurred related to OTSC system. Complications just occurred on 2 (0.3%) patients after application OTSC system. One case occurred on a duodenal ulcer bleeding patient who experienced a tiny GI leak which was suspected to be caused by the clip. Another adverse event was lumen obstruction after a duodenal OTSC application. None of these studies reported severe and fatal complication. Twelve studies reported the mortality rate was 10.9% (*n* = 84), which related to re-bleeding or continued bleeding was 3.9% (*n* = 30) and due to other causes were 7.0% (*n* = 54).

### Quality of included studies

Table 3 showed the quality scores of each study according to the Downs and Black checklist. Five studies scored low methodological quality (score range 8 to 9), 8 studies scored low to moderate quality (score range 10 to 14), two study scored moderate-quality (score range 15 to 17), and one study which was randomized trial scored 20 considered as high-quality.

**Table 3:** Quality assessment of included studies

Study	Downs checklist	Methodological quality
Kirschniak et al. [16]	9	Low
Albert et al. [17]	9	Low
Manta et al. [18]	10	Moderate/low
Mönkemüller et al. [19]	8	Low
Chan et al. [11]	10	Moderate/low
Skinner et al. [12]	10	Moderate/low
Manno et al. [20]	10	Moderate/low
Wedi et al. [21]	9	Low
Richter-Schrag et al. [22]	15	Moderate
Lamberts et al. [23]	11	Moderate/low
Goenka et al. [24]	9	Low
Wedi et al. [25]	12	Moderate/low
Schmidt et al. [26]	20	High
Asokkumar et al. [27]	14	Moderate/low
Manta et al. [13]	14	Moderate/low
Gölder et al. [28]	17	Moderate

### Publication Bias

**Fig. 7:**
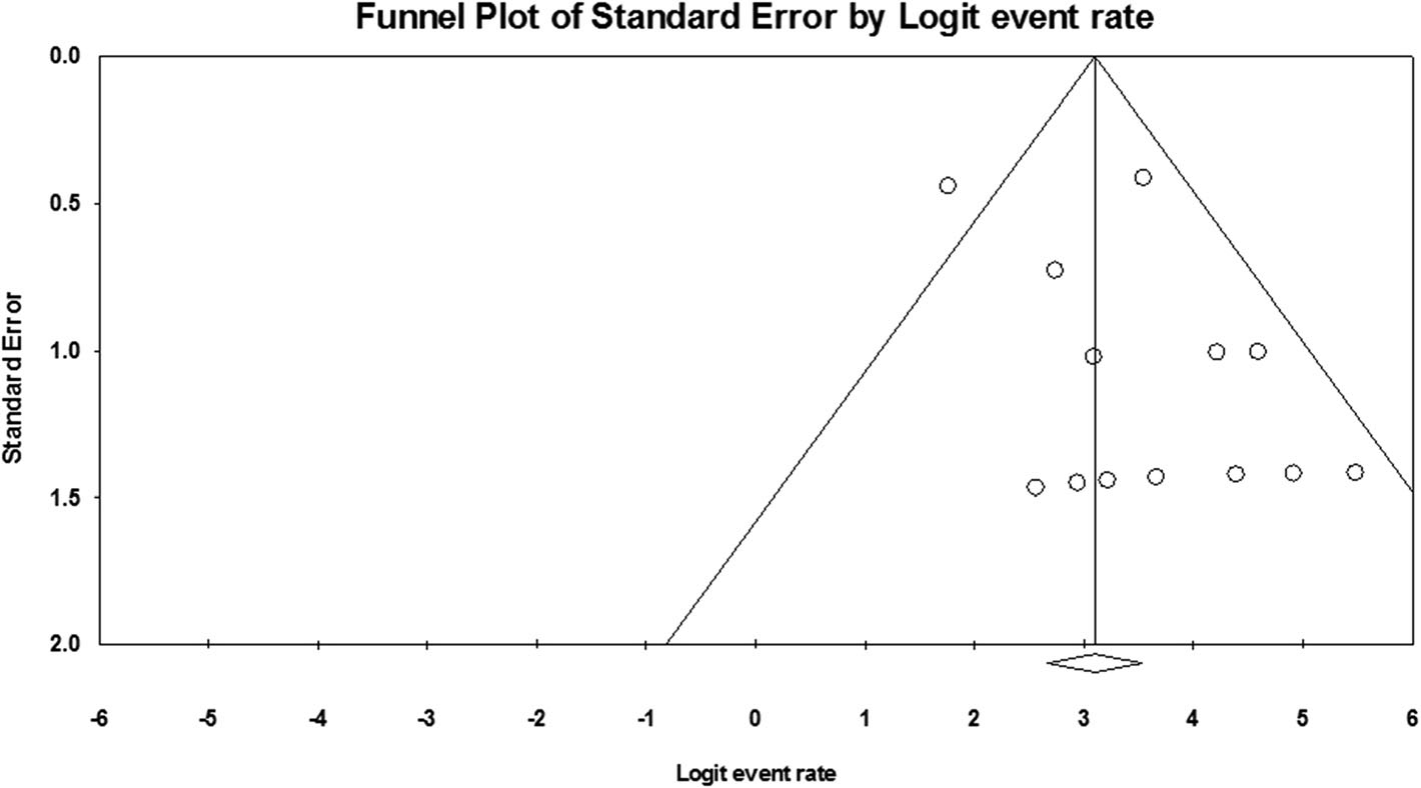
Funnel plot for publication bias of technical success

**Fig. 8:**
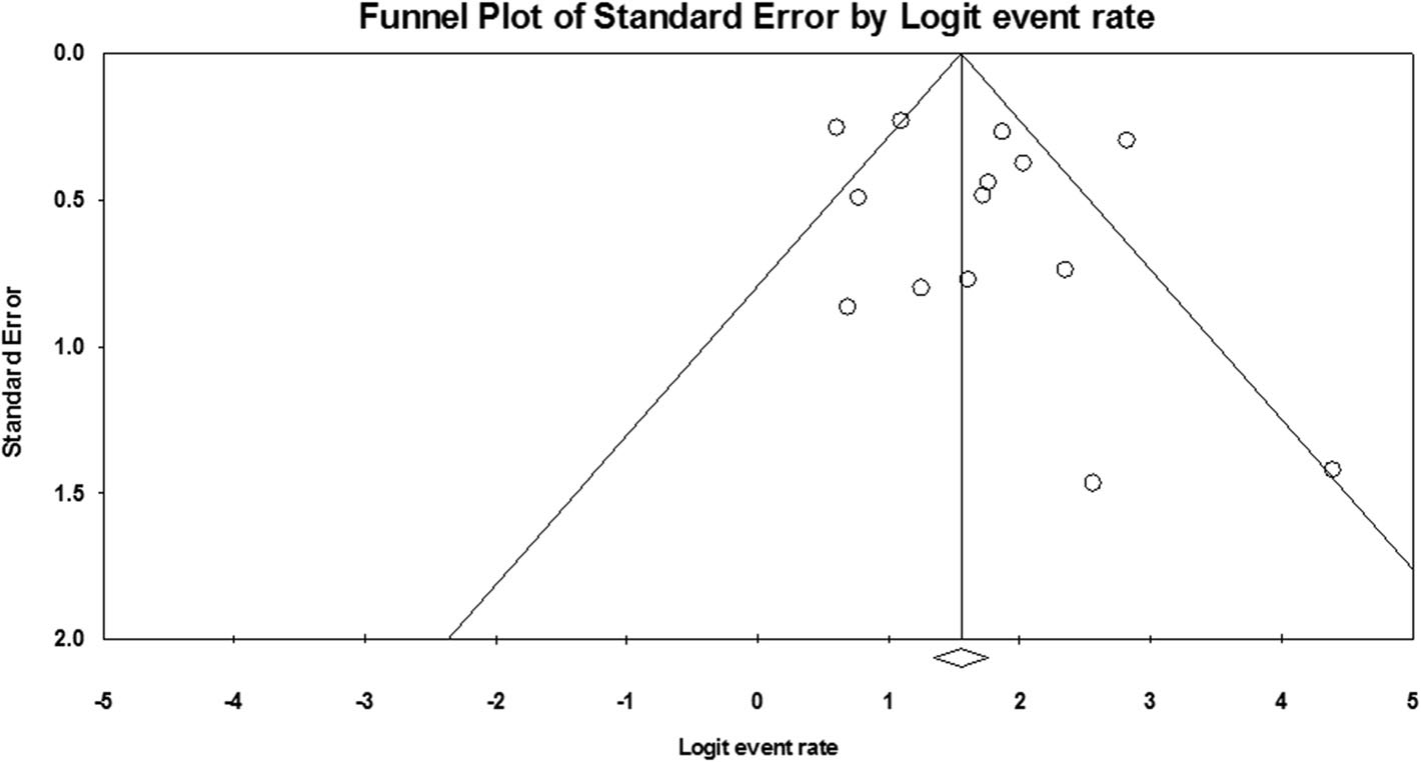
Funnel plot for publication bias of clinical success

Funnel plot of technical success and clinical success was demonstrated in Fig. 7 and Fig. 8. Through visual inspection of the funnel plot, no publication bias can be generally considered. By Egger’s test, technical success rate (*P* = 0.123) and clinical success rate (*P* = 0.346) had no significant publication bias.

## Discussion

UNVGIB is a common and potentially life-threatening emergency. Currently, endoscopic hemostatic treatment has been a gold standard therapy for UNVGIB, which included endoscopic hemoclips, thermal therapy and injection therapy, alone or in combination with each other [20]. However, the total re-bleeding rate after endoscopic treatment was reported to 5–20% [29–31]. So it is urgent to develop a more effective endoscopic device or technique for UNVGIB. Recently, the OTSC system, as novel management for GI bleeding, has drawn great attention in clinical practice. The OTSC system is a full-thickness suturing device designed for flexible endoscopy. It can be used to close the wound surface of target diseases, such as non-variceal GI bleeding, perforation, fistula, and anastomotic dehiscence [32]. At present, a number of studies reported the usefulness of OTSC system for UNVGIB. In this study, we combined the data from these reports, which allowed us to provide the best evidence on the effectiveness and safety of OTSC system for UNVGIB.

Our study demonstrated that the OTSC system was a technically feasible and effective (95.7% technical success rate and 84.2% clinical success rate) modality in achieving hemostasis of UNVGIB. The OTSC system was relatively safe with a tiny minority adverse events. The high clinical success rate and minimal complication profile indicated that the OTSC system was a viable method for patients with UNVGIB.

There was just one comparative study in our systematic review, which aimed to compare standard endoscopic treatment versus OTSC system [26]. It was demonstrated that OTSC system was associated with greater efficacy and lower re-bleeding rate when compared to standard therapy with through-the-scope clips in patients with recurrent peptic ulcer bleeding after successful initial hemostasis. So the authors favored the use of OTSC system for patients with recurrent bleeding of peptic ulcers. But we still can not reach the conclusion that OTSC system was superior to other endoscopic treatments for recurrent bleeding because of the limited data in our study.

Recently, a systematic review by Ofosu et al. [33] reported a total of 16 studies which involved 475 non-variceal gastrointestinal bleeding patients treated with OTSC. In their report, the hemostasis rate achieved with primary application of OTSC was 93% (95% CI, 89–96%). Similarly, the hemostasis rate achieved with rescue OTSC therapy was 91% (95% CI, 84–95%). Re-bleeding rates after primary OTSC therapy were 21% (95% CI, 8–43%) and 25% (95% CI, 17–34%) with rescue OTSC therapy. Our results were quite close to their report. We found the OTSC system applied as the primary treatment modality in 507 lesions, while 190 lesions as rescue treatment modality after previous endoscopic treatment failure. And most of those patients we included were at high risk of re-bleeding. Five of 15 studies emphasized the OTSC system could reduce significantly re-bleeding rates and mortality in high-risk cohort and could be a first-line treatment for UNVGIB [13, 20, 22, 25, 27]. Conversely, some authors concluded that OTSC system might be considered as a secondary option for high-risk patients after conventional endoscopic hemostasis failed [11, 12, 22, 23]. Therefore, OTSC may be considered as first-line treatment for UNVGIB in high risk patients and rescue treatment for initial failed hemostasis with conventional endoscopic methods.

Seven of 16 studies in our systematic review reported the reasons for OTSC treatment failure: 1) delayed closure of OTSC occurring in lesions with large caliber artery and those with deep fibrotic base; 2) shallow placement of OTSC resulting from inadequate suction or premature clip deployment; and 3) misplacement of OTSC because of poor visualization, difficult anatomy, and unstable endoscope position [11, 12, 18, 22, 23, 27, 28]. These failure causes of OTSC above may enlighten its further application in future.

The safety of OTSC system for management of UNVGIB should be carefully assessed. Our study revealed that adverse events were rare. Just 2 of 16 studies reported 2 patients experienced the complications. Albert et al. reported one patient with duodenal oozing ulcer experienced a tiny GI leak which was suspected to be caused by the OTSC system, then the patients were converted to the surgical department [17]. Richter-Schrag et al. reported lumen obstruction after a duodenal OTSC application. Then, the obstruction released with 3 balloon-dilatations [22]. In addition, there were other OTSC-related complications reported in some studies when OTSC was applied for closure GI perforation or fistula, such as esophageal perforation, acute cholangitis, inadvertent tongue piercing and jejunal stenosis [34–37]. Our study showed 10.9% of patients died during the follow-up time. Although this data was surprising, only 3.9% patient died related to OTSC system application failure and most of these patients died due to fatal comorbidity. Gölde et al. deemed that in case of severe recurrent bleeding, the bleeding source could be controlled by endoscopic treatment, but the patient refused any further therapy and died, which was one of the reasons for the high mortality [28].

While our study suggested a promising role of OTSC system for UNVGIB, further consideration is warranted regarding cost. In addition, clinical expertise and unfamiliarity among endoscopists in the small center may factor into variable technical and clinical success results. The expense of this modality for UNVGIB as well as availability remains unclear in our included studies. Future studies are needed to truly assess the cost-effectiveness of OTSC system placement for the management of UNVGIB.

Certainly, we recognized that some limitations in our study. First, our systematic review and meta-analysis were based completely and only on the published literature. We could not get the data of the individual patient, which would allow us to perform more detailed analysis, such as subgroup analysis of OTSC for different etiologies of bleeding. Second, some included studies were missing data for our review variables of interest, such as bleeding classification, lengths of hospital stay and additional therapy modality. Finally, just one literature was high methodological quality study, and our review was lack of comparing OTSC system to other therapy modality.

## Conclusions

Our study demonstrated that the OTSC system was a technically feasible modality and highly efficacious in achieving hemostasis in acute UNVGIB. It is a promising endoscopic technique with high success rate and the rare adverse event. In the future, more randomized controlled trials are needed to compare OTSC to other therapy modality.

## Supplementary information

**Additional file 1: Table S1.** Detailed search terms of each of search engines used in analysis.

## Data Availability

Not applicable.

## Abbreviations

CI: Confidence interval
GI: Gastrointestinal
OTSC: Over-the-scope-clip
SD: Standard deviation
UNVGIB: Upper non-variceal gastrointestinal bleeding
