# Supplementary material for: Clinical outcomes of over-the-scope-clip system for the treatment of acute upper non-variceal gastrointestinal bleeding: a systematic review and meta-analysis
Source: BMC Gastroenterol. 2019 Dec 23;19:225. doi: 10.1186/s12876-019-1144-4 (PMC6929512; doi:10.1186/s12876-019-1144-4)
Supplement: Supplementary file 1 — Additional file 1: Table S1. Detailed search terms of each of search engines used in analysis. [file 12876_2019_1144_MOESM1_ESM.doc]

Table S1 Detailed search terms of each of search engines used in analysis.

| PubMed | (OTSC OR OTSC system OR over the scope OR over the scope clip OR over-the-scope clip OR OVESCO OR Ovesco clip OR hemoclips)  AND  (Gastrointestinal Hemorrhage [MeSH Terms] OR Hemorrhage, Gastrointestinal OR Gastrointestinal Hemorrhages OR Hematochezia OR Hematochezias OR gastrointestinal bleeding OR gastrointestinal bleed OR gastrointestinal hemorrhage OR GI bleeding OR GI bleed OR GI hemorrhage OR bleeding OR bleed OR hemorrhage OR ulcer bleeding OR ulcer hemorrhage OR hemostasis OR melena)  NOT  (variceal gastrointestinal bleeding) |
| --- | --- |
| Embase | (“OTSC”:ab,ti OR “OTSC system”:ab,ti OR “over the scope”:ab,ti OR “over the scope clip”:ab,ti OR “over-the-scope clip”:ab,ti OR “OVESCO”:ab,ti OR “Ovesco clip”:ab,ti OR “hemoclips”:ab,ti)  AND  (“Gastrointestinal Hemorrhage”:ab,ti OR “Hemorrhage, Gastrointestinal”:ab,ti OR “Gastrointestinal Hemorrhages”:ab,ti OR “Hematochezia”:ab,ti OR “Hematochezias”:ab,ti OR “gastrointestinal bleeding”:ab,ti OR “gastrointestinal bleed”:ab,ti OR “gastrointestinal hemorrhage”:ab,ti OR “GI bleeding”:ab,ti OR “GI bleed”:ab,ti OR “GI hemorrhage”:ab,ti OR “bleeding”:ab,ti OR “bleed”:ab,ti OR “hemorrhage”:ab,ti OR “ulcer bleeding”:ab,ti OR “ulcer hemorrhage”:ab,ti OR “hemostasis”:ab,ti OR “melena”:ab,ti) |
| Cochrane | (OTSC OR OTSC system OR over the scope OR over the scope clip OR over-the-scope clip OR OVESCO OR Ovesco clip OR hemoclips) |
